# A SOX9‐AS1/miR‐5590‐3p/SOX9 positive feedback loop drives tumor growth and metastasis in hepatocellular carcinoma through the Wnt/β‐catenin pathway

**DOI:** 10.1002/1878-0261.12560

**Published:** 2019-08-31

**Authors:** Wei Zhang, Yanhui Wu, Bingwu Hou, Yadong Wang, Dongfeng Deng, Zhihao Fu, Zongquan Xu

**Affiliations:** ^1^ Hepatic Surgery Center Tongji Hospital Tongji Medical College Huazhong University of Science and Technology Wuhan Hubei China; ^2^ Department of Hepatobiliary Pancreatic Surgery Henan Provincial People's Hospital, People's Hospital of Zhengzhou University People's Hospital of Henan University Henan China

**Keywords:** hepatocellular carcinoma, microRNA‐5590‐3p, SOX9, SOX9‐AS1, Wnt/β‐catenin pathway

## Abstract

Hepatocellular carcinoma (HCC) is a prevalent solid tumor with a high global death rate. SRY box 9 (SOX9) has been reported as an oncogene in HCC by several studies, but the underlying mechanism remains largely unexplored. Here, we confirmed upregulation of SOX9 in HCC tissues and cell lines and validated that SOX9 facilitates proliferation, migration and invasion in HCC. We subsequently identified that the long non‐coding RNA (lncRNA) SOX9 antisense RNA 1 (SOX9‐AS1) is a neighbor gene to SOX9; SOX9‐AS1 is also upregulated in HCC, and its expression is positively correlated with that of SOX9. In addition, SOX9‐AS1 appears to have prognostic significance in HCC patients. We showed that SOX9‐AS1 aggravates HCC progression and metastasis *in vitro* and *in vivo*. We demonstrated that SOX9‐AS1 sponges miR‐5590‐3p to elevate SOX9 expression, and that SOX9 in turn transcriptionally activates SOX9‐AS1. Moreover, we verified that SOX9‐AS1 regulates SOX9 and its known downstream Wnt/β‐catenin pathway so as to facilitate epithelial‐to‐mesenchymal transition. The results of our rescue assays suggest that SOX9‐AS1 regulates HCC progression through SOX9 and the Wnt/β‐catenin pathway. In conclusion, our study demonstrates that a SOX9‐AS1/miR‐5590‐3p/SOX9 positive feedback loop drives tumor growth and metastasis in HCC through the Wnt/β‐catenin pathway, suggesting SOX9‐AS1 as a novel potential prognostic and treatment target for HCC.

**Abbreviations**
ChIP: chromatin immunoprecipitation
DAPI: 4′,6‐diamidino‐2‐phenylindole
EMT: epithelial‐to‐mesenchymal transition
FISH: RNA fluorescence *in situ* hybridization
HCC: hepatocellular carcinoma
IF: immunofluorescence
IG: immunoglobulin G
IHC: immunohistochemistry
LIHC: liver hepatocellular carcinoma
lncRNA: long non‐coding RNA
miRNA: microRNA
NC: negative control
RIP: RNA immunoprecipitation
RT‐qPCR: quantitative real‐time polymerase chain reaction
SOX9‐AS1: SOX9 antisense RNA 1
SOX9: SRY box 9
TCGA: The Cancer Genome Atlas
TF: transcription factor
WT: wild type

## 1 Introduction

Hepatocellular carcinoma (HCC) is a solid tumor prevalent around the globe and is recognized as the second leading cause of tumor‐associated mortality in China (Thomas *et al*., 2010). Although the therapeutic strategies for HCC have been upgraded, the 5‐year survival rate in HCC patients is still dismal, owing to a major extent to the high recurrence rate and easy metastasis following surgical dissection (Aldrighetti *et al*., 2009; Yang *et al*., 2016). Therefore, a better understanding of the molecular mechanism related to HCC progression and metastasis is needed.

Sex‐determining region Y (SRY) related high‐mobility group box 9 (SOX9) belongs to the SRY box gene superfamily (Foster *et al*., 1994). It is known to be a transcription factor (TF) and has oncogenic roles in human cancers (Afonja *et al*., 2002; Ling *et al*., 2011; Shi *et al*., 2018). Moreover, the role of SOX9 in HCC has been illustrated in several studies. For instance, SOX9 has been reported to be associated with progression and poor prognosis in HCC (Guo *et al*., 2012). MicroRNA (miRNA)‐138 suppressed HCC cell by inhibiting SOX9 (Liu *et al*., 2016). Interestingly, SOX9 has been reported to regulate β‐catenin expression positively in the canonical Wnt pathway and to influence the downstream effectors such as Cyclin D1 and c‐Myc to promote cancer progression (Santos *et al*., 2016). The regulation of SOX9 on the Wnt/β‐catenin pathway has been reported in several cancers such as lung cancer (Cui *et al*., 2017; Guo *et al*., 2018) and HCC (Leung *et al*., 2016). However, the mechanism of SOX9 in HCC still needs to be explored further.

Long non‐coding RNA (lncRNA) are RNA transcripts longer than 200 nucleotides possessing no obvious protein‐coding ability (Schmitt and Chang, 2016). The lncRNA can exert regulatory functions in multiple biological processes such as differentiation, development and tumorigenesis (Balci *et al*., 2016; Lü *et al*., 2015; Ma *et al*., 2016). To date, the dysregulation and impact of lncRNA in HCC have been reported in HCC in a number of studies. For example, lncRNA CARLo‐5 facilitated HCC progression by inhibiting miR‐200b (Dou *et al*., 2017), and lncRNA UBE2CP3 promoted epithelial‐to‐mesenchymal transition (EMT) to induce metastasis in HCC (Cao *et al*., 2017). We were interested in SOX9 antisense RNA 1 (SOX9‐AS1) because it is a nearby gene to SOX9, and The Cancer Genome Atlas (TCGA) database showed its upregulation in HCC. It has been revealed that lncRNA potentially regulates its nearby genes (Jiang *et al*., 2019; Yan *et al*., 2017). Therefore, we speculated that SOX9‐AS1 might be related to SOX9 and participate in HCC. The association of SOX9‐AS1 with HCC and SOX9 has never been explored before.

It has been largely reported that lncRNA could serve as a competitive endogenous RNA to regulate target genes. For example, in HCC, lncRNA NNT‐AS1 promoted progression and metastasis by regulating the miR‐363/CDK6 axis (Lu *et al*., 2017). LINC00052 regulated EPB41L3 by targeting miR‐452‐5p to inhibit HCC (Zhu *et al*., 2017). The miRNA are conserved non‐coding transcripts with about 22 nucleotides, responsible for silencing gene expression through targeting 3′UTR of mRNA (Bartel, 2009; Brümmer and Hausser, 2014). The miR‐5590‐3p is a newly identified miRNA exhibiting inhibitive function in gastric cancer through targeting DDX5/AKT/mTOR pathway (Wu *et al*., 2018), but its involvement in HCC and correlation with SOX9‐AS1 and SOX9 have rarely been reported.

The present study aimed to explore the mechanism of SOX9 in HCC.

## 2 Materials and methods

### 2.1 Tissue collection

Hepatocellular carcinoma tissue samples (*n* = 67) and the paired adjacent non‐cancerous tissue samples (*n* = 67) were collected. All patients signed informed consents, and the protocols were approved by the Research Ethics Committee of Tongji Hospital, Tongji Medical College, Huazhong University of Science and Technology. Patients underwent surgical resection without radiotherapy or chemotherapy at the Tongji Hospital, Tongji Medical College, Huazhong University of Science and Technology. All samples were snap‐frozen immediately in liquid nitrogen and kept at −80 °C immediately after being dissected, and all samples were validated by two experienced pathologists. The study methodologies conformed to the standards set by the Declaration of Helsinki.

### 2.2 Cell culture

Human HCC cell lines (Huh7, HepG2, HCCLM3 and Hep3B) and the normal liver cell lines (L02) obtained from Cell Bank of the Shanghai Branch of Chinese Academy of Sciences (Shanghai, China) were cultured in Dulbecco's modified Eagle's medium (Gibco; Thermo Fisher Scientific, Inc., Waltham, MA, USA) supplemented with 10% FBS (HyClone, South Logan, UT, USA) and 1% penicillin/streptomycin (Gibco). The culture conditions were 37 °C with 5% CO_2_.

### 2.3 Cell transfection

SOX9‐AS1‐specific short hairpin RNA (shRNA) (sh‐SOX9‐AS1#1, sh‐SOX9‐AS1#2 and sh‐SOX9‐AS1#3), SOX9‐specific shRNA (sh‐SOX9#1, sh‐SOX9#2 and sh‐SOX9#3) and negative control (sh‐NC) were produced by RiboBio (Guangzhou, China). The full‐length sequences of SOX9‐AS1 or SOX9 were integrated into the pcDNA3.1 vector (Invitrogen, Carlsbad, CA, USA) to generate pcDNA3.1/SOX9‐AS1 and pcDNA3.1/SOX9 (termed SOX9‐AS1 and SOX9) according to the manufacturer's instructions. The overexpression and knockdown of miR‐5590‐3p were realized by miR‐5590‐3p mimic and miR‐5590‐3p inhibitor, with NC mimic and NC inhibitor as controls. Cell transfections were performed with Lipofectamine 2000 (Invitrogen) according to the manufacturers’ instructions.

### 2.4 Quantitative RT‐PCR (RT‐qPCR)

Total RNA was isolated from HCC cells using Trizol reagent (Invitrogen), followed by the generation of complementary DNA (cDNA) through reverse transcription by High Capacity cDNA Reverse Transcription Kit (Applied Biosystems, Darmstadt, Germany). The RT‐PCR was accomplished using SYBR Green real‐time PCR kit (TaKaRa, Dalian, China). GAPDH and U6 were internal references for normalization of mRNA and miRNA expressions. The calculation of expression was performed using the $2^{-\Delta \Delta C_{t}}$ method. All the primers were shown as follows:

SOX9‐AS1: forward, 5′‐ACGTCAGCGAGCTTGAGAAA‐3′, reverse, 5′‐GACATACGTCGGGAGCTCAG‐3′;

SOX9: forward, 5′‐AGGTGCTCAAAGGCTACGACTG‐3′, reverse, 5′‐CCTAATGTTCATGGTCGGCGC‐3′;

GAPDH: forward, 5′‐CCCATCACCATCTTCCAGGAG‐3′, reverse 5′‐GTTGTCATGGATGACCTTGGC‐3′;

U6: forward, 5′‐CTCGCTTCGGCAGCACA‐3′, reverse, 5′‐AACGCTTCACGAATTTGCGT‐3′.

### 2.5 Cell Counting Kit 8 (CCK‐8)

Cell Counting Kit‐8 (CCK‐8; Dojindo Molecular Technologies, Inc., Kumamoto, Japan) was used to examine cell proliferation, following the manufacturers’ protocol. A 10‐μL aliquot of CCK‐8 reagent was added after HCC cells plated in 96‐well plates (1 × 10^3^ cells per well) were cultured for 0, 24, 48, 72 and 96 h, and the cells then incubated for a further 2 h at 37 °C. Then, optical density (wavelength: 450 nm) was determined by a microplate reader (Model 550; Bio‐Rad Laboratories, Inc., Hercules, CA, USA).

### 2.6 Colony formation assay

HCC cells 5 × 10^2^ were incubated in each well of six‐well plates. Following the incubation at 37 °C for 14 days, colonies generated by HCC cells were subjected to fixation with 4% formaldehyde and staining with 0.25% crystal violet. Colonies formed of more than 50 cells were counted by a microscope.

### 2.7 Cell invasion and migration assays

Cell migration was determined via wound‐healing assay. The ‘wound’ was artificially created on the confluent cell monolayer. The scratches were treated with mitomycin C (10 μg·mL^−1^) for 2 h. The wound at 0 and 24 h was observed by inverted microscope (Olympus, Tokyo, Japan). The ratio of wound closure after 24 h incubation was calculated to measure cell migration.

To determine cell invasion, HCC cells in serum‐free medium were plated into the upper chamber of the Transwell (8‐μm pore; BD Biosciences, San Jose, CA, USA) pre‐coated with 1 mg·mL^−1^ Matrigel (BD Biosciences). Medium containing 10% FBS was added to the lower chamber. HCC cells that had invaded were fixed by methanol and then stained with crystal violet. A microscope was used to observe and count the cells from five randomly chosen fields.

### 2.8 Luciferase reporter assay

Luciferase assays were performed using the luciferase reporter assay system (Promega, Madison, WI, USA) according to the manufacturers’ instructions. To evaluate the interaction of miR‐5590‐3p with SOX9‐AS1 and SOX9, a SOX9‐AS1 and SOX9 3′‐UTR sequence containing the miR‐5590‐3p sites or mutated sites was generated into the pmirGLO vector (Promega) to construct WT‐SOX9‐AS1, Mut‐SOX9‐AS1, WT‐SOX9 and Mut‐SOX9. The above‐mentioned reporter plasmids were respectively co‐transfected with miR‐5590‐3p mimics or NC mimics into 293T cells. To evaluate the promoter activity of SOX9, the promoter sequence of SOX9 was inserted into the pmirGLO vector and co‐transfected with sh‐SOX9‐AS1#1, sh‐SOX9‐AS1#2 or SOX9‐AS1, with sh‐NC or pcDNA3.1 as control, into 293T cells. To evaluate the promoter activity of SOX9‐AS1, the wild type (WT) promoter sequence of SOX9‐AS1 containing predicted binding sites (E1 and E2) was inserted into the pmirGLO vector to generate WT reporter. The Mut (E1), Mut (E2) or Mut (E1 + E2) reporters (with E1, E2 or both sites mutated) were constructed as well. The plasmids above were respectively co‐transfected with pcDNA3.1 or SOX9 into 293T cells. All transfections were conducted with Lipofectamine 2000 (Invitrogen). After transfection for 2 days, the luciferase activities were detected by the dual luciferase assay (Promega), with Renilla luciferase activities as normalized control.

To evaluate the activity of Wnt/β‐catenin, the β‐catenin TOP‐flash reporter and FOP‐flash were transfected with Renilla TK‐luciferase vector (pRL‐TK; Promega) into HCC cells. Following 2 days of transfection, luciferase activity was measured by the Dual Luciferase Reporter Assay System (Promega). Firefly luciferase activity was normalized to Renilla luciferase activity, and β‐catenin‐mediated transcription was determined by TOP/FOP ratio.

### 2.9 RNA fluorescence *in situ* hybridization (FISH)

Briefly, HCC cells fixed in 4% formaldehyde underwent pepsin treatment and dehydration using EtOH. HCC cells were incubated with the FISH probe of SOX9‐AS1 (RiboBio) in hybridization buffer for 1 h. Following hybridization, the slides were subjected to washing and dehydration. The cells were then mounted with ProLong® Gold Antifade Reagent with the addition of 4′,6‐diamidino‐2‐phenylindole (DAPI; Cell Signaling Technology, Danvers, MA, USA) for detection. The slides were observed with an Olympus microscope FV1200 (Olympus).

### 2.10 Immunofluorescence (IF)

Hepatocellular carcinoma cells fixed in 4% formaldehyde were treated with pepsin and subjected to dehydration through EtOH. After permeabilization in Triton X‐100 (Sigma‐Aldrich Co., St. Louis, MO, USA), samples were blocked by goat serum and then incubated with anti‐E‐cadherin and anti‐N‐cadherin (Abcam, Cambridge, MA, USA) overnight at 4 °C. After washing off primary antibodies, cells were incubated for 1 h with appropriate secondary antibody. Cell nuclei were then stained with DAPI. The visualization was conducted using a fluorescence microscope (DMI4000B; Leica Microsystems, Wetzlar, Germany).

### 2.11 RNA immunoprecipitation (RIP) assay

RNA immunoprecipitation assay was performed to evaluate the interaction of miR‐5590‐3p with SOX9‐AS1 and SOX9 using the EZ‐Magna RIP RNA‐binding protein immunoprecipitation kit (Millipore, Billerica, MA, USA), following the manufacturer's instructions. In brief, HCC cells reaching 80–90% confluency were lysed by the RIP lysis buffer, followed by the incubation with RIP buffer with magnetic beads that were conjugated with anti‐argonaute 2 (Ago2; Millipore). The NC was isotype‐matched immunoglobulin G (IgG). The eluted RNA were subjected to analysis by RT‐qPCR.

### 2.12 Chromatin immunoprecipitation (ChIP)

Chromatin immunoprecipitation was done according to the manufacturer's protocol for the ChIP Assay Kit (Millipore, Temecula, CA, USA). Briefly, the chromatin DNA was cross‐linked with 1% formaldehyde, separated by sonication, and immunoprecipitated with anti‐SOX9 (Sigma‐Aldrich), with IgG as NC. Thereafter, immunoprecipitated DNA was subjected to elution and analyzed by RT‐qPCR with the primers designed according to the promoter sequences of SOX9‐AS1 containing wild‐type binding sites for SOX9, mutated binding site 1 [Mut (E1)], mutated binding site 2 [Mut (E2)] or mutated binding sites 1 and 2 [Mut (E1 + E2)].

### 2.13 Western blot analysis

After protein extraction from HCC cells in radioimmunoprecipitation lysis buffer (Invitrogen), 10% SDS/PAGE was used to separate equal amounts of proteins, followed by the shifting of proteins to polyvinylidene difluoride membranes (Millipore Corporation, Billerica). The membranes were then detected with primary antibodies of SOX9, β‐catenin, Cyclin D1, c‐Myc, E‐cadherin, N‐cadherin (Abcam, UK) and GAPDH (Santa Cruz Biotechnology Inc., Dallas, TX, USA). Thereafter, blots were incubated with the horseradish peroxidase‐conjugated secondary antibody (Abcam). Visualization of blots was conducted by the standard enhanced chemiluminescence procedure (Millipore, Billerica). Analysis of signals was performed on image lab software (Bio‐Rad Laboratories, Inc.).

### 2.14 Tumor xenograft model in nude mice

Animal experiments were conducted to examine the role of SOX9‐AS1 *in vivo* with the approval of the Institutional Animal Care Committee of Tongji Hospital, Tongji Medical College, Huazhong University of Science and Technology. Male BALB/C nude mice 4–6 weeks old were purchased from Slac Laboratory Animal Center (Shanghai, China) and kept under the specific pathogen‐free conditions. Mice were housed in micro‐isolator cages and provided with food and water, following the Guide for the Care and Use of Laboratory Animals. Mice were then injected subcutaneously with the suspension of Huh7 cells (3 × 10^6^·100 mL^–1^) with the transfection of sh‐SOX9‐AS1#1 or sh‐NC. Tumor volume was determined every 7 days. Tumor volume was evaluated based on the following equation: 0.5 × length × width^2^. After 28 days, tumors were resected for further detection.

### 2.15 Immunohistochemistry (IHC)

After the dewaxed paraffin‐embedded tissue sections were subjected to rehydration, the samples were placed into citrate buffer (10 mm, pH 6.0) and heated twice in a microwave oven. Sections were incubated for 10 min with 3% H_2_O_2_ and washed in PBS. After being blocked by 10% normal goat serum for 0.5 h, samples were incubated with the purified anti‐Ki67, anti‐PCNA, anti‐E‐cadherin, anti‐N‐cadherin and normal control IgG at 4 °C for 12 h. Following washing, each section was stained using a catalyzed sign amplification system kit (Dako code k5007) and observed by looking at photos of the sections.

### 2.16 Statistical analysis

All experiments were conducted three times. Data were expressed as mean ± standard deviation (SD). Overall survival was analyzed by Kaplan–Meier analysis and log‐rank test. Spearman's correlation analysis was utilized to determine the linear correlations between two variables. Two‐group differences were examined by Student's *t*‐test, and multi‐group differences were examined by one‐way ANOVA. *P* < 0.05 was used to determine statistical significance.

## 3 Results

### 3.1 SOX9 was upregulated in HCC and aggravated cell proliferation, migration, invasion and EMT

**Figure 1:**
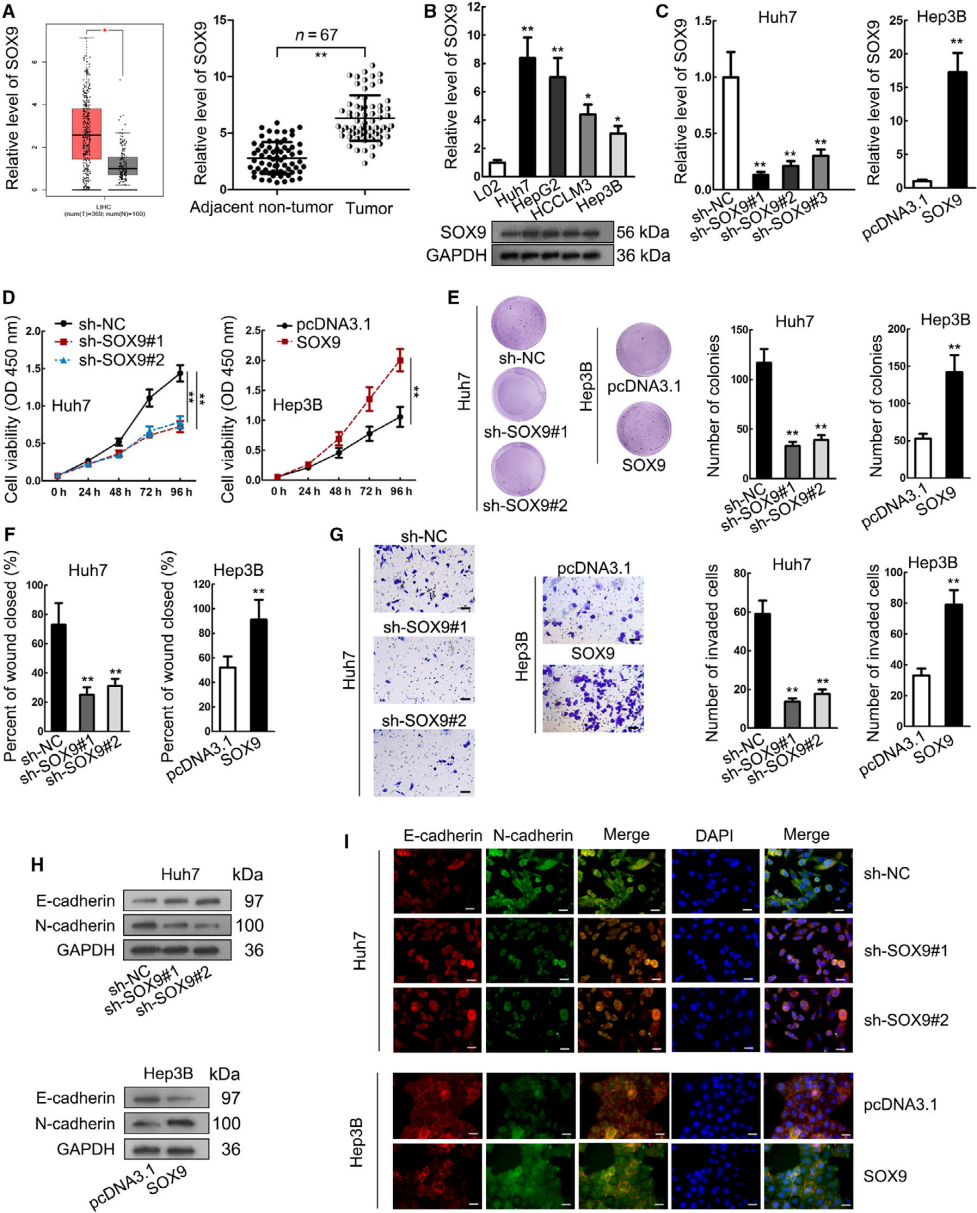
SOX9 was upregulated in HCC and aggravated cell proliferation, migration, invasion and EMT. (A) SOX9 upregulation in LIHC in TCGA database and RT‐qPCR results of SOX9 upregulation in HCC samples. (B) RT‐qPCR and western blot analyses of SOX9 upregulation in HCC cells at mRNA and protein levels. (C) SOX9 knockdown by sh‐SOX9#1, sh‐SOX9#2 or sh‐SOX9#3 in Huh7 cells and SOX9 overexpression by pcDNA3.1/SOX9 in Hep3B cells were confirmed by RT‐qPCR. (D, E) CCK‐8 and colony formation assays were used to assess proliferation of HCC cells upon SOX9 knockdown and overexpression. (F, G) Wound‐healing assay and Transwell invasion assay were used to assess HCC cell migration and invasion upon SOX9 knockdown and overexpression. Scale bar: 50 μm. (H, I) Western blot and IF staining were used to assess expressions of N‐cadherin and E‐cadherin in HCC cells upon SOX9 knockdown and overexpression. Scale bar: 25 μm. *P < 0.05, **P < 0.01. Significance was determined by Student's *t*‐test. Error bars indicate SD. All experiments were performed three times.

First, we examined the involvement of SOX9 in HCC. TCGA data presented the upregulation of SOX9 in liver hepatocellular carcinoma (LIHC) samples (Fig. 1A, left) and we confirmed the high expression of SOX9 in HCC tissues by RT‐qPCR analysis (Fig. 1A, right). The mRNA and protein levels of SOX9 were also elevated in HCC cell lines compared with normal cell line (Figs 1B and S2). Next, we investigated the function of SOX9 in HCC. Since SOX9 was confirmed to exhibit the highest expression in Huh7 cells and the lowest expression in Hep3B cells, we silenced SOX9 in Huh7 cells and overexpressed SOX9 in Hep3B cells for loss‐ and gain‐of‐function assays. RT‐qPCR data validated the knockdown and overexpression of SOX9, and we found that sh‐SOX9#1 and sh‐SOX9#2 were the most efficient for SOX9 knockdown (Fig. 1C). Hence, the subsequent loss‐of‐function assays were conducted with sh‐SOX9#1 and sh‐SOX9#2. We observed that the silenced expression of SOX9 in Huh7 cells impeded cell proliferation, whereas forced expression of SOX9 in Hep3B cells had the opposite effect (Fig. 1D,E). In addition, migration and invasion of HCC cells were weakened by SOX9 knockdown, whereas this enhanced by SOX9 overexpression (Fig. 1F,G). Additionally, since the facilitated EMT progression allowed stronger migratory and invasive ability of tumor cells (Thiery *et al*., 2009), we measured the effect of SOX9 on the expressions of EMT markers. Consequently, expression of the epithelial marker E‐cadherin increased in the presence of SOX9 knockdown and decreased in the presence of SOX9 overexpression, whereas expression of mesenchymal marker N‐cadherin presented the opposite result (Figs 1H, I and S2). The above‐mentioned data suggested that SOX9 was upregulated in HCC and promoted cell proliferation, migration, invasion and EMT.

### 3.2 SOX9‐AS1 was upregulated in HCC and positively regulated SOX9 to regulate Wnt/β‐catenin pathway and EMT

**Figure 2:**
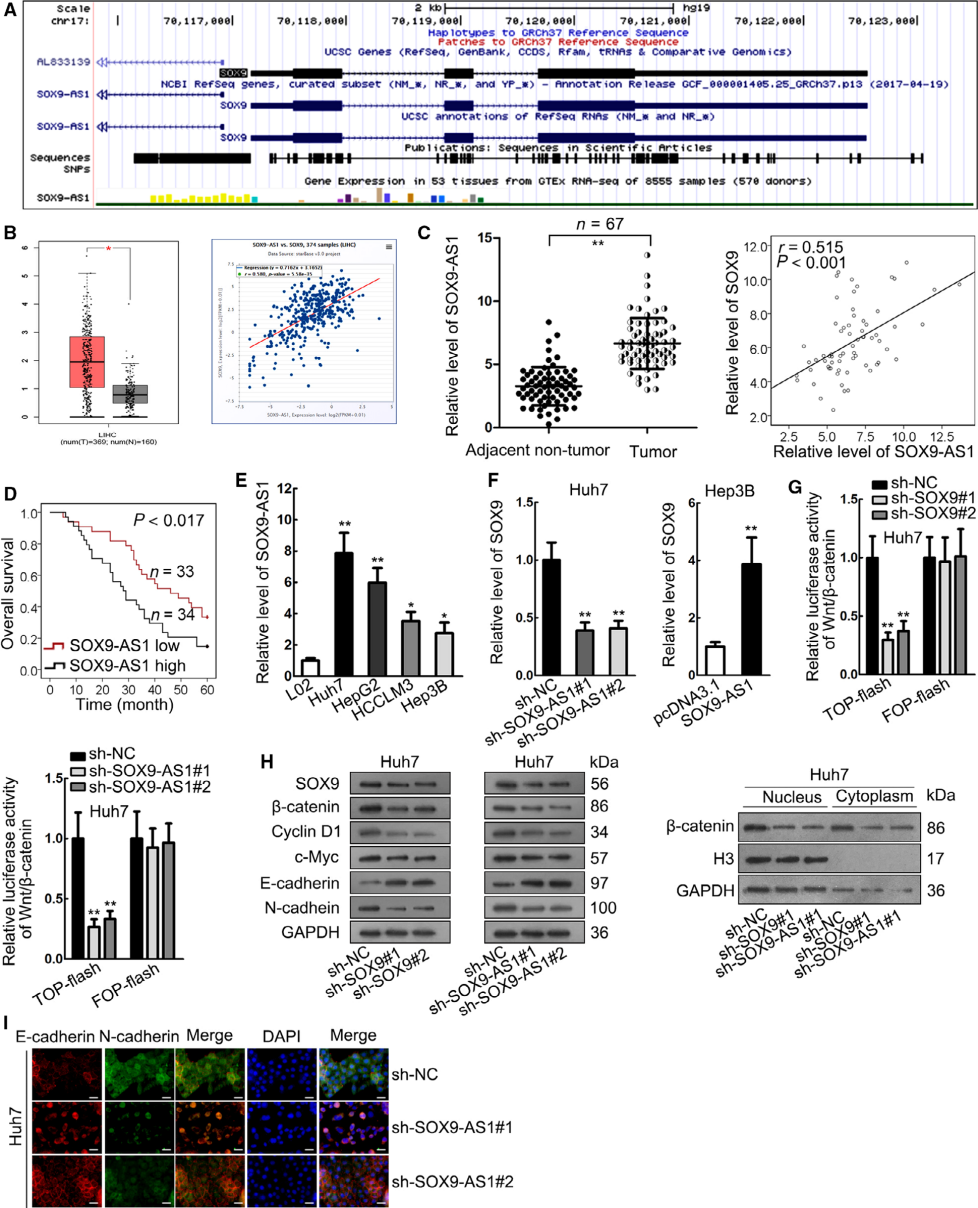
SOX9‐AS1 positively regulated SOX9 to regulate Wnt/β‐catenin pathway and EMT. (A) SOX9‐AS1 was identified as a nearby gene for SOX9 in UCSC database. (B) TCGA data showed the upregulation of SOX9‐AS1 and the positive correlation between SOX9‐AS1 and SOX9 in LIHC samples. (C) RT‐qPCR analysis confirmed the upregulation of SOX9‐AS1 in HCC tissues and Spearman's correlation analysis confirmed the positive correlation between SOX9‐AS1 and SOX9 in HCC tissues. (D) Kaplan–Meier analysis and log‐rank test identified the prognostic significance of SOX9‐AS1 in HCC patients. (E) RT‐qPCR results of the upregulation of SOX9‐AS1 in HCC cell lines. (F) RT‐qPCR results of SOX9 expression under knockdown and overexpression of SOX9‐AS1. (G) TOP‐flash assay was used to evaluate the activity of Wnt/β‐catenin pathway with silencing of SOX9 and SOX9‐AS1. (H) Western blot results of the expressions of SOX9, β‐catenin, Cyclin D1, c‐Myc, N‐cadherin and E‐cadherin with silencing of SOX9 and SOX9‐AS1. The expression of β‐catenin in both nucleus and cytoplasm was reduced by SOX9‐AS1 and SOX9. (I) IF staining results of the expressions of N‐cadherin and E‐cadherin with silencing of SOX9‐AS1. Scale bar: 25 μm. *P < 0.05, **P < 0.01. Significance was determined by Student's *t*‐test. Error bars indicate SD. All experiments were performed three times.

We tried to figure out the mechanism underlying SOX9 upregulation in HCC. Increasing studies have shown that lncRNA are functional in regulating gene expression in human cancers (Liu *et al*., 2017) and that they potentially regulate their nearby genes (Jiang *et al*., 2019; Yan *et al*., 2017). By searching UCSC (http://genome.ucsc.edu/) we found that lncRNA SOX9‐AS1 is the neighbor of SOX9 (Fig. 2A), indicating that SOX9‐AS1 might have a relation with SOX9. To find out whether SOX9‐AS1 participates in HCC, we first analyzed its expression in TCGA database through the GEPIA (http://gepia.cancer-pku.cn/) and Starbase pan‐cancer (http://starbase.sysu.edu.cn/panCancer.php) online analysis tools. We discovered that SOX9‐AS1 was upregulated in LIHC samples (Fig. 2B, left) and positively correlated with SOX9 expression in LIHC (Fig. 2B, right). We also confirmed these results in HCC samples (Fig. 2C). Kaplan–Meier analysis demonstrated that high SOX9‐AS1 expression was an indicator of low overall survival in HCC patients (Fig. 2D). Thereafter, we verified the upregulation of SOX9‐AS1 in HCC cell lines (Fig. 2E). Moreover, to test the effect of SOX9‐AS1 on SOX9 expression, we silenced SOX9‐AS1 in Huh7 cells and overexpressed it in Hep3B cells (Fig. S1A). SOX9 expression was reduced by SOX9‐AS1 knockdown and was induced by SOX9‐AS1 overexpression (Fig. 2F). Previously, SOX9 has been reported to positively regulate β‐catenin expression in the canonical Wnt pathway and downstream effectors such as Cyclin D1 and c‐Myc for regulating cancer progression (Santos *et al*., 2016). Furthermore, a former study has demonstrated that SOX9 can regulate Wnt/β‐catenin pathway to promote stemness in HCC (Leung *et al*., 2016). The Wnt/β‐catenin pathway has been largely reported as crucial signaling in the regulation of progression and metastases in HCC (Chao *et al*., 2017; Hu *et al*., 2018; Zhao *et al*., 2017). Therefore, we wondered whether SOX9 and SOX9‐AS1 could regulate the Wnt/β‐catenin pathway in HCC. TOP‐flash assay showed that silencing SOX9 or SOX9‐AS1 could reduce the activity of the Wnt/β‐catenin pathway (Fig. 2G). Western blot analysis showed that silencing of SOX9‐AS1 or SOX9 caused a decrease in the expression of SOX9, β‐catenin, Cyclin D1, c‐Myc and N‐cadherin, and an increase in the expression of E‐cadherin (Figs 2H and S2). Additionally, we confirmed through western blot that silencing SOX9 or SOX9‐AS1 could decrease the expression of β‐catenin in both cytoplasm and nucleus (Fig. 2H). IF staining revealed the increase of E‐cadherin and the decrease of N‐cadherin in response to SOX9‐AS1 knockdown (Fig. 2I). These results indicated that SOX9‐AS1 was upregulated in HCC, and positively regulated SOX9 to activate the Wnt/β‐catenin pathway and EMT.

### 3.3 SOX9‐AS1 aggravated cell proliferation, migration and invasion in HCC

**Figure 3:**
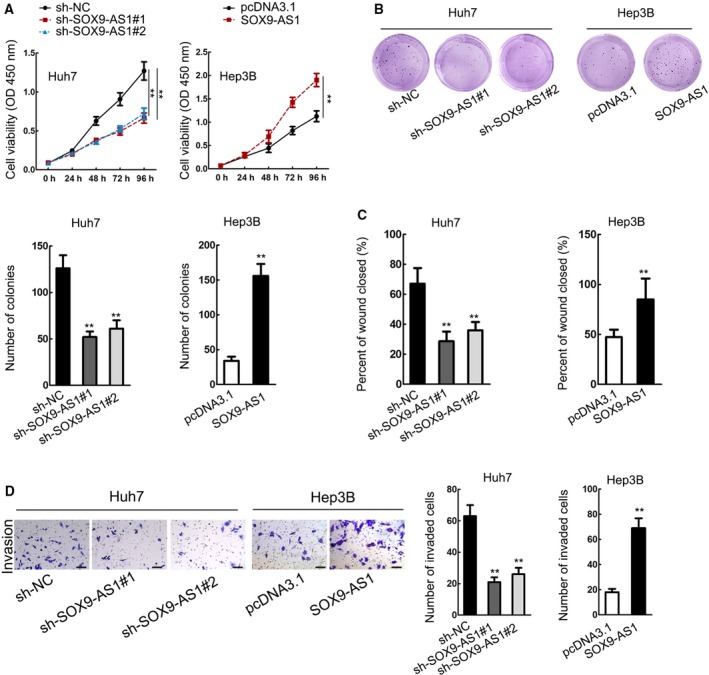
SOX9‐AS1 aggravated cell proliferation, migration and invasion in HCC. (A, B) CCK‐8 and colony formation assays were used to assess proliferation of HCC cells on SOX9‐AS1 knockdown and overexpression. (C, D) Wound‐healing assay and Transwell invasion assay were used to assess HCC cell migration and invasion on SOX9‐AS1 knockdown and overexpression. Scale bar: 50 μm. **P < 0.01. Significance was determined by Student's *t*‐test. Error bars indicate SD. All experiments were performed three times.

We examined the function of SOX9‐AS1 in HCC by loss‐ and gain‐of‐function assays. It was observed that SOX9‐AS1 depletion hampered proliferation in Huh7 cells, whereas SOX9‐AS1 overexpression prompted proliferation in Hep3B cells (Fig. 3A,B). The migration and invasion of HCC cells were attenuated by SOX9‐AS1 silence but were facilitated by SOX9‐AS1 overexpression (Fig. 3C,D). Collectively, these data indicated that SOX9‐AS1 aggravated cell proliferation, migration and invasion in HCC.

### 3.4 SOX9‐AS1 sponged miR‐5590‐3p to induce SOX9/Wnt/β‐catenin

**Figure 4:**
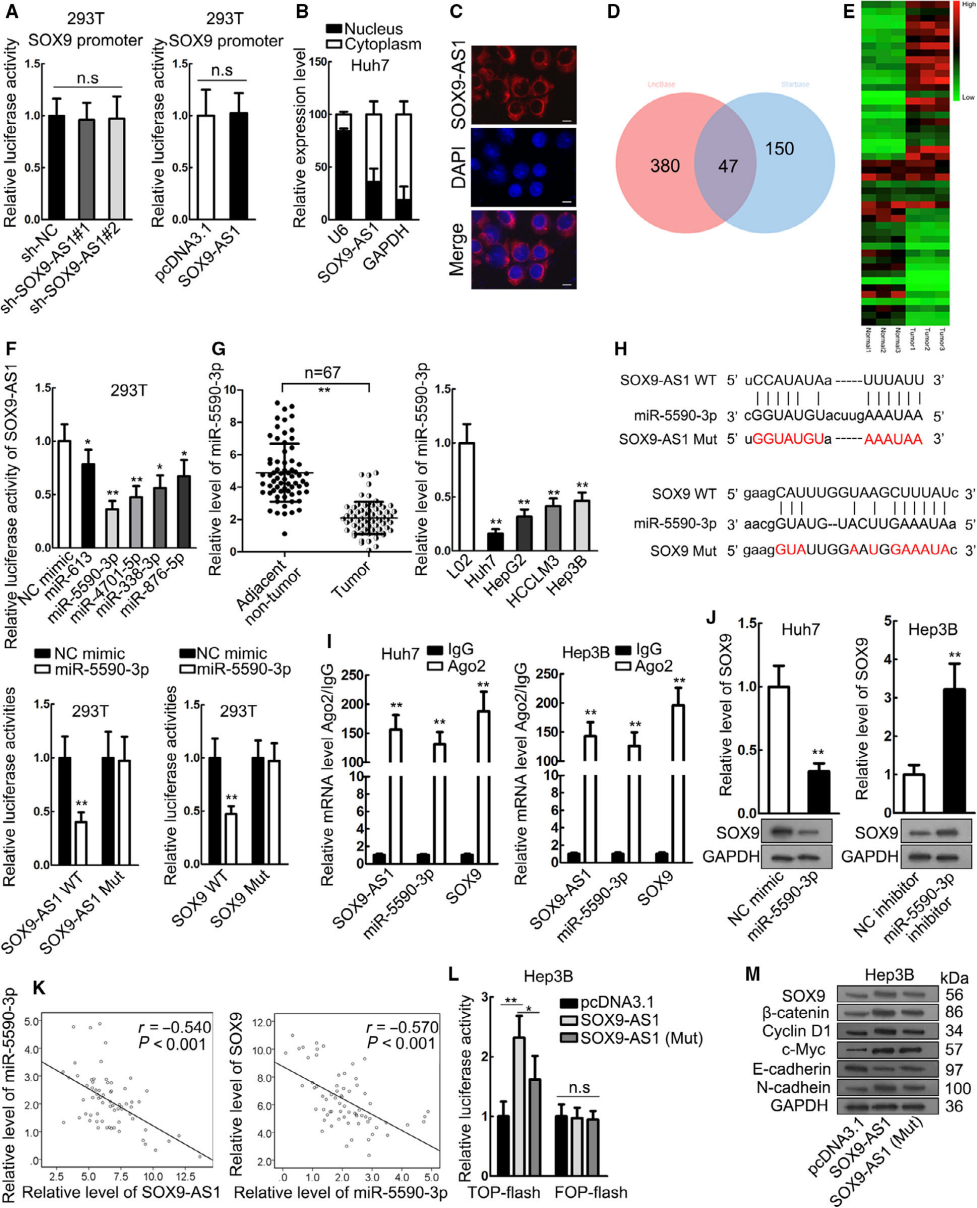
SOX9‐AS1 sponged miR‐5590‐3p to induce SOX9/Wnt/β‐catenin. (A) Luciferase activity assay validated the non‐effect of SOX9‐AS1 on SOX9 promoter. (B, C) Subcellular fractionation and FISH staining confirmed that SOX9‐AS1 was mainly expressed in cytoplasm. Scale bar: 10 μm. (D) The intersection of Starbase and LncBase results showed 47 miRNA potentially interacting with both SOX9 and SOX9‐AS1. (E) RT‐qPCR results of the 47 miRNA in three pairs of HCC tissues and matched adjacent non‐cancerous tissues; the top five downregulated miRNA were miR‐613, miR‐5590‐3p, miR‐4701‐5p, miR‐338‐3p and miR‐876‐5p. (F) Luciferase reporter assay showed that overexpression of miR‐5590‐3p led to the most significant reduction of SOX9‐AS1 reporter. (G) RT‐qPCR results of the downregulation of miR‐5590‐3p in HCC tissues and cell lines. (H) The binding sites on SOX9‐AS1 and SOX9 3′UTR for miR‐5590‐3p and the mutant sites. Luciferase reporter analysis was used to determine the interaction of miR‐5590‐3p with SOX9‐AS1 and SOX9. (I) RIP assay confirmed the interaction of miR‐5590‐3p with SOX9‐AS1 and SOX9. (J) RT‐qPCR and western blot analyses of SOX9 expression on miR‐5590‐3p overexpression and knockdown. (K) Spearman's correlation analysis showed the negative correlation of miR‐5590‐3p with SOX9‐AS1 and SOX9. (L) TOP‐flash assay was used to assess the activity of the Wnt/β‐catenin pathway upon overexpression of wild‐type SOX9‐AS1 or SOX9‐AS1 with mutant miR‐5590‐3p sites. (M) Western blot analysis of the expression of SOX9, β‐catenin, Cyclin D1, c‐Myc, N‐cadherin and E‐cadherin upon overexpression of wild‐type SOX9‐AS1 or SOX9‐AS1 with mutant sites for miR‐5590‐3p. *P < 0.05, **P < 0.01. Significance was determined by Student's *t*‐test or one‐way ANOVA. Error bars indicate SD. All experiments were performed three times.

We explored how SOX9‐AS1 regulated SOX9 in HCC. First, we found that neither knockdown nor overexpression SOX9‐AS1 caused any significant change in the luciferase activity of SOX9 promoter reporter (Fig. 4A), indicating that SOX9‐AS1 might regulate SOX9 post‐transcriptionally. We then identified that SOX9‐AS1 was primarily expressed in cytoplasm of HCC cells (Fig. 4B,C). It has been reported that lncRNA could regulate target gene expression by acting as an miRNA sponge in cytoplasm of HCC cells (Li *et al*., 2017; Lu *et al*., 2017), so we speculated that SOX9‐AS1 might regulate SOX9 through targeting miRNA. We identified 380 candidate miRNA for SOX9‐AS1 through LncBase (http://carolina.imis.athena-innovation.gr/diana_tools/web/index.php?r=lncbasev2/index-predicted) and 150 candidate miRNA targeting SOX9 through Starbase (http://starbase.sysu.edu.cn/), and 47 miRNA were identified to potentially interact with both SOX9‐AS1 and SOX9 (Fig. 4D). To further narrow the candidates, we detected the expressions of 47 miRNA in three pairs of HCC tissues and the matched para‐tumorous tissues. Consequently, we picked the five most downregulated miRNA in HCC tissues – miR‐613, miR‐5590‐3p, miR‐4701‐5p, miR‐338‐3p and miR‐876‐5p (Fig. 4E). Luciferase reporter assay demonstrated that among the five miRNA, overexpression of miR‐5590‐5p led to the most significant alleviation of luciferase activity on SOX9‐AS1 reporter (Fig. 4F), indicating that miR‐5590‐3p had the strongest interaction with SOX9‐AS1. We therefore focused on miR‐5590‐3p. We validated the downregulation of miR‐5590‐3p in HCC tissues and cell lines (Fig. 4G). Thereafter, we investigated the interaction of miR‐5590‐3p with SOX9‐AS1 and SOX9. The binding sites on SOX9‐AS1 and SOX9 3′UTR are presented in Fig. 4H. Luciferase reporter assay showed that overexpressing miR‐5590‐3p reduced the luciferase activity of SOX9‐AS1 WT and SOX9 WT, instead of SOX9‐AS1 Mut and SOX9 Mut (Fig. 4H). RIP‐qPCR analysis confirmed the enrichment of SOX9‐AS1, SOX9 and miR‐5590‐3p in Ago2 precipitates (Fig. 4I). Moreover, the influence of miR‐5590‐3p on SOX9 expression was determined. We overexpressed miR‐5590‐3p in Huh7 cells and inhibited it in Hep3B cells (Fig. S1B). SOX9 mRNA and protein expression were decreased by miR‐5590‐3p overexpression, whereas they were increased by miR‐5590‐3p inhibition (Figs 4J and S2). Spearman's correlation analysis confirmed that miR‐5590‐3p was negatively correlated with SOX9‐AS1 and SOX9 in HCC tissues (Fig. 4K). Additionally, we found that overexpressing SOX9‐AS1 with mutant binding sites for miR‐5590‐3p had a less powerful effect than overexpressing wild type SOX9‐AS1 on the activation of Wnt/β‐catenin (Fig. 4L). Also, the effect of SOX9‐AS1 (Mut) was less powerful than SOX9‐AS1 upon increasing the expression of SOX9, β‐catenin, Cyclin D1, c‐Myc, and N‐cadherin and decreasing the expression of E‐cadherin (Figs 4M and S2). The partial reverse caused by mutating miR‐5590‐3p sites revealed by TOP‐flash assay and western blot assay above indicated that other miRNA might also be involved in the regulation of SOX9‐AS1 on SOX9/Wnt/β‐catenin. Altogether, the data above suggested that SOX9‐AS1 sponged miR‐5590‐3p to induce SOX9/Wnt/β‐catenin.

### 3.5 SOX9 transcriptionally induced SOX9‐AS1 in HCC

**Figure 5:**
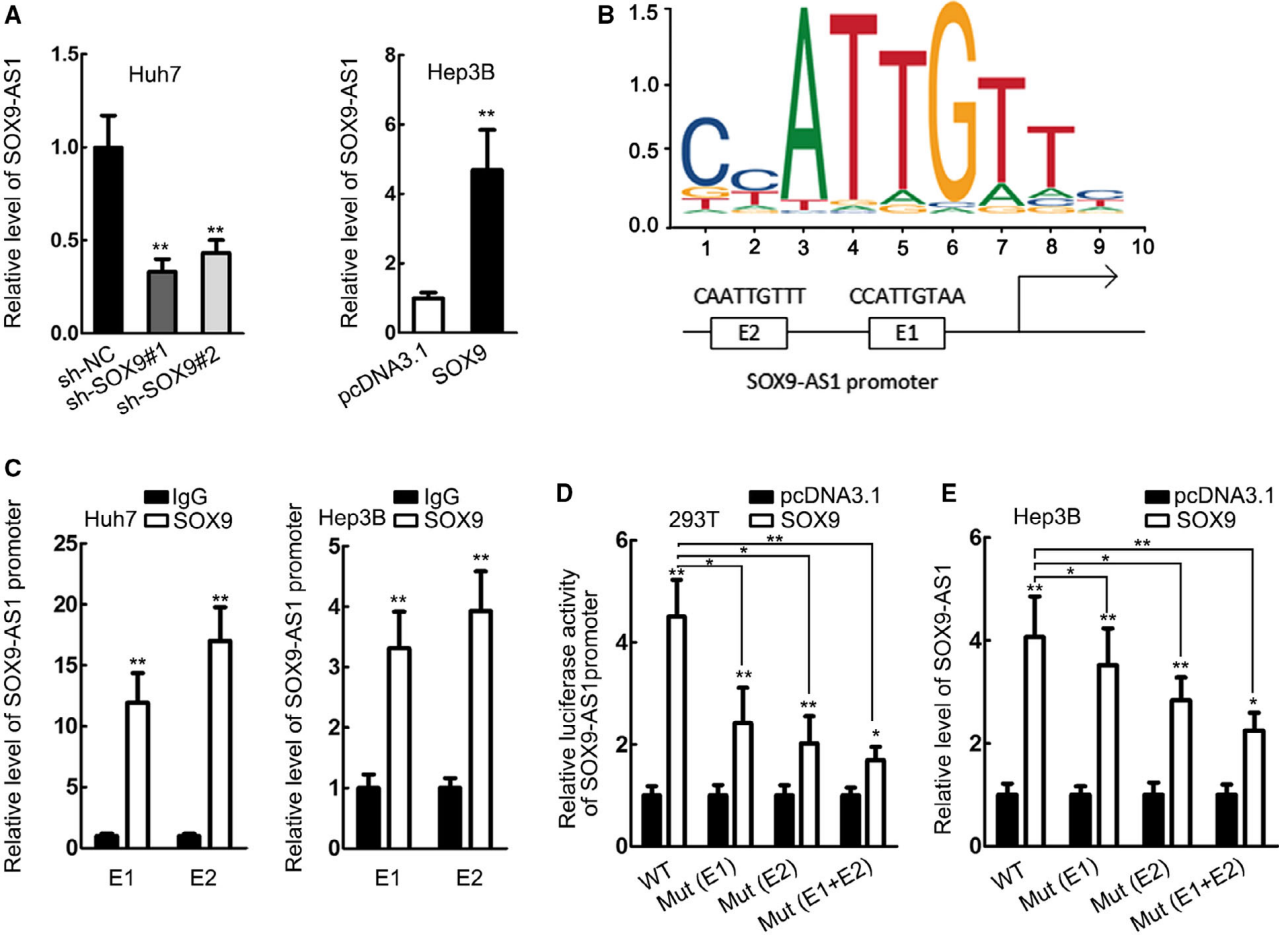
SOX9 transcriptionally induces SOX9‐AS1 in HCC. (A) RT‐qPCR results of SOX9‐AS1 expression upon knockdown and overexpression of SOX9. (B) The DNA motif of SOX9 and the binding sites on SOX9‐AS1 promoter for SOX9 (relative score > 9) was obtained using the JASPAR tool. (C, D) ChIP and luciferase reporter assays confirmed the binding of SOX9 to SOX9‐AS1 on the specific sites. (E) The expression of SOX9‐AS1 with wild‐type sites, mutant site 1, mutant site 2 or mutant site 1 and 2. *P < 0.05, **P < 0.01. Significance was determined by Student's *t*‐test. Error bars indicate SD. All experiments were performed three times.

Since SOX9 is known as a TF (Shi *et al*., 2018) and former report has determined that SOX9 is responsible for the upregulation of lncRNA MALAT1 in lung cancer (Chen *et al*., 2017), we asked whether SOX9 could transcriptionally regulate SOX9‐AS1 expression in HCC. We found that silencing SOX9 resulted in reduction of SOX9‐AS1 expression, whereas overexpressing SOX9 had the opposite effect (Fig. 5A). To investigate the interaction between SOX9 and SOX9‐AS1, we applied the JASPAR tool (http://jaspar.genereg.net/). By comparing the binding motif of SOX9 and promoter sequence of SOX9‐AS1 in the JASPAR tool, we identified two binding sites (relative score > 0.9) on SOX9‐AS1, termed E1 and E2 (Fig. 5B). ChIP analysis revealed that both E1 and E2 were abundant in the binding complex of SOX9 (Fig. 5C). Luciferase activity showed that mutating either E1 or E2 could weaken the inductive effect of SOX9 overexpression on SOX9‐AS1 promoter activity, and mutating both E1 and E2 could further aggravate such a result (Fig. 5D). The same results were also observed in the expression of SOX9‐AS1 expression (Fig. 5E). Therefore, it was indicated that SOX9 transcriptionally induced SOX9‐AS1 in HCC.

### 3.6 SOX9‐AS1 regulated HCC progression through SOX9/Wnt/β‐catenin pathway

**Figure 6:**
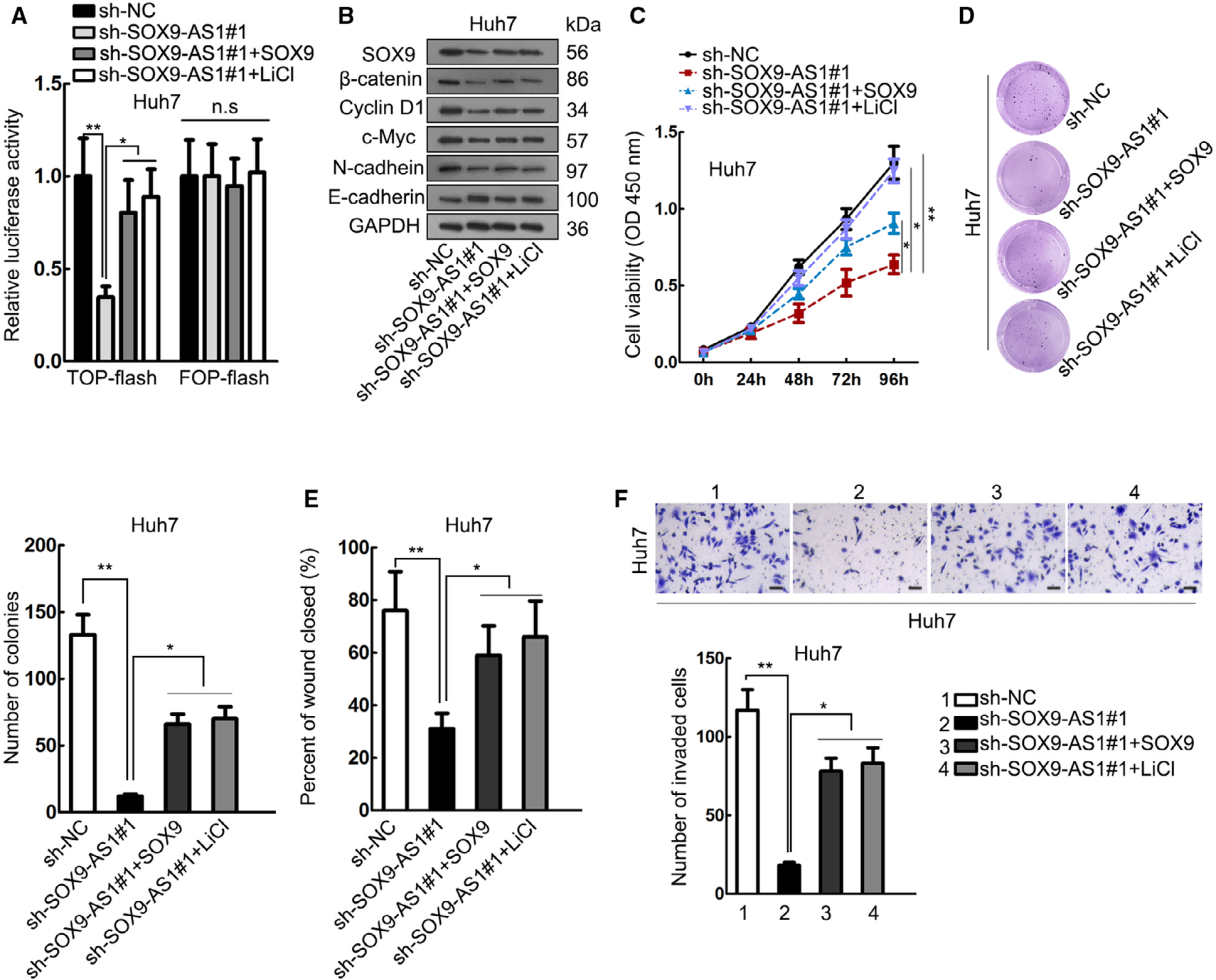
SOX9‐AS1 regulated HCC progression through the SOX9/Wnt/β‐catenin pathway. Huh7 cells were treated with sh‐NC, sh‐SOX9‐AS1#1, sh‐SOX9‐AS1#1 + SOX9, and sh‐SOX9‐AS1#1+LiCl, respectively. (A) TOP‐flash assay was used to assess the activity of the Wnt/β‐catenin pathway of each group. (B) Western blot results of the expression of SOX9, β‐catenin, Cyclin D1, c‐Myc, N‐cadherin and E‐cadherin of each group. (C, D) CCK‐8 and colony formation assays were used to evaluate HCC cell proliferation of each group. (E, F) Wound‐healing assay and Transwell invasion assay were used to assess HCC cell migration and invasion of each group. Scale bar: 50 μm. *P < 0.05, **P < 0.01. Significance was determined by Student's *t*‐test or one‐way ANOVA. Error bars indicate SD. All experiments were performed three times.

Subsequently, we performed rescue assays to investigate whether SOX9‐AS1 regulated HCC progression through the SOX9/Wnt/β‐catenin pathway. We found that overexpression of SOX9 or treatment with LiCl (the activator of Wnt/β‐catenin) could rescue the activity of Wnt/β‐catenin inhibited by SOX9‐AS1 silencing (Fig. 6A). Western blot assay results depicted that the decreased expression of SOX9, β‐catenin, Cyclin D1, c‐Myc and N‐cadherin, and the increased expression of E‐cadherin upon SOX9‐AS1 knockdown, could be reversed by either SOX9 overexpression or LiCl treatment (Figs 6B and S2). The inhibitive effect of SOX9‐AS1 knockdown on cell proliferation, migration and invasion could be countervailed by SOX9 overexpression or Wnt/β‐catenin activation (Fig. 6C–F). In summary, SOX9‐AS1 regulated HCC proliferation, migration, invasion and EMT through the SOX9/Wnt/β‐catenin pathway.

### 3.7 SOX9‐AS1 drove tumor growth and metastasis of HCC *in vivo*

**Figure 7:**
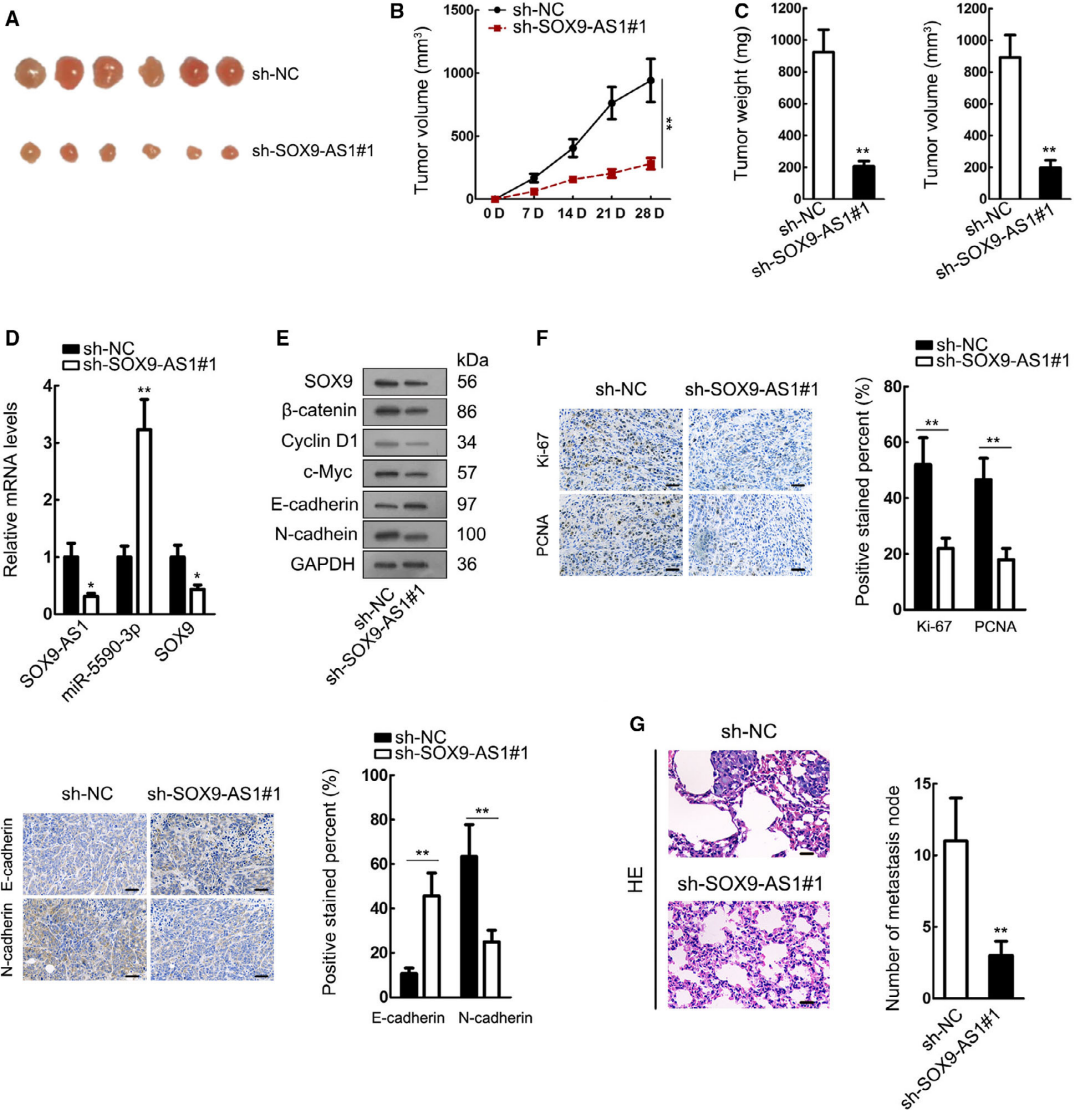
SOX9‐AS1 drove tumor growth and metastasis of HCC *in vivo*. Huh7 cells transfected with sh‐NC or sh‐SOX9‐AS1#1 were injected into nude mice to generate xenografts. (A) Pictures of tumors from mice of each group. (B) The growth curve of xenografts in mice of each group. (C) The final weight and volume of tumors from mice of each group. (D) RT‐qPCR results of expression of SOX9‐AS1, miR‐5590‐3p and SOX9 of tumors from mice of each group. (E) Western blot analysis of SOX9, β‐catenin, Cyclin D1, c‐Myc, N‐cadherin and E‐cadherin expressions in tumors of each group. (F) IHC staining of Ki67, PCNA, E‐cadherin and N‐cadherin in tumors from mice of each group. Scale bar: 50 μm. (G) HE staining results of metastatic nodes in mice of each group. Scale bar: 50 μm.*P < 0.05, **P < 0.01. Significance was determined by Student's *t*‐test. Error bars indicate SD. All experiments were performed three times.

Finally, we carried out animal experiments to examine the effect of SOX9‐AS1 *in vivo*. Huh7 cells transfected with sh‐NC or sh‐SOX9‐AS1 were inoculated into nude mice and the tumor growth was determined over time. Silencing SOX9‐AS1 led to the generation of smaller tumors in mice (Fig. 7A). The growth curve of xenografts in mice suggested that SOX9‐AS1 knockdown retarded tumor growth *in vivo* (Fig. 7B). After 28 days, the tumors were resected and it was observed that tumors of mice with SOX9‐AS1 silencing presented a reduction on tumor weight and volume (Fig. 7C). We confirmed in the xenografts that SOX9‐AS1 knockdown reduced SOX9‐AS1 and SOX9 expression and induced miR‐5590‐3p expression (Fig. 7D). Data from western blot analysis confirmed that SOX9‐AS1 depletion reduced the protein levels of SOX9, β‐catenin, Cyclin D1, c‐Myc and N‐cadherin, and increased the level of E‐cadherin *in vivo* (Fig. 7E). IHC staining revealed the lessened positivity of proliferation markers (Ki67 and PCNA) and N‐cadherin, and the increased positivity of E‐cadherin in the tumors from SOX9‐AS1‐silenced mice (Figs 7F and S2). Moreover, we observed that mice with SOX9‐AS1 knockdown had fewer metastatic nodes (Fig. 7G). In conclusion, SOX9‐AS1 drove tumor growth and metastasis of HCC *in vivo*.

## 4 Discussion

In spite of the upgrading of treatment tools for HCC, the outcome for patients with HCC is still a gloomy one, which calls for a further understanding of the mechanism behind HCC progression. Studies have revealed that the dysregulated gene is involved in cancer regulation (Guo *et al*., 2012). SOX9 is a TF in the SRY box gene superfamily (Foster *et al*., 1994). Previous studies have demonstrated the high expression and prognostic significance of SOX9 in HCC and the oncogenic function of SOX9 in tumor progression in HCC (Guo *et al*., 2012; Liu *et al*., 2016). These findings suggested that SOX9 was a crucial regulator in HCC and that a better understanding of the SOX9‐related mechanism in HCC might be helpful in improving treatments for HCC. In concordance with previous reports, our study validated that SOX9 was upregulated in HCC tissues in both TCGA data and collected patient samples, and that SOX9 facilitated proliferation, migration, invasion and EMT in HCC *in vitro*.

Former studies illustrated that lncRNA could regulate certain gene expressions in human cancers, and SOX9 has been revealed to be regulated in several lncRNA in cancers such as lncRNA MALAT1 and SNHG1 (Chen *et al*., 2017; Cui *et al*., 2017). However, the regulation of SOX9 expression by lncRNA in HCC is rarely explored. Previous studies have indicated that lncRNA potentially regulate their nearby genes (Jiang *et al*., 2019; Yan *et al*., 2017), so we searched for nearby lncRNA for SOX9 in the UCSC database. Interestingly, we found a new lncRNA SOX9‐AS1. We identified the upregulation of SOX9‐AS1 and its positive correlation with SOX9 in LIHC samples from TCGA database and confirmed this result in HCC tissues and cells. We also confirmed that SOX9‐AS1 suggested a poor outcome in HCC patients. Furthermore, we verified the positive regulation of SOX9‐AS1 on SOX9 expression. Additionally, several studies have reported that SOX9 could regulate the Wnt/β‐catenin pathway in human cancers (Cui *et al*., 2017; Guo *et al*., 2018; Santos *et al*., 2016), including HCC (Leung *et al*., 2016). The Wnt/β‐catenin pathway has been recognized as a key pathway implicated in HCC progression and metastasis (Chao *et al*., 2017; Hu *et al*., 2018; Zhao *et al*., 2017). Herein, we showed that silencing SOX9 or SOX9‐AS1 could attenuate the Wnt/β‐catenin pathway, thereby inhibiting EMT. Functionally, we revealed that SOX9‐AS1 promoted proliferation, migration and invasion of HCC.

Mechanistically, lncRNA could regulate target genes at transcriptional and post‐transcriptional level. In our study, the non‐effect of SOX9‐AS1 on the SOX9 promoter and the cytoplasmic expression of SOX9‐AS1 indicated that SOX9‐AS1 might regulate SOX9 at the post‐transcriptional level. An increasing number of studies have revealed that lncRNA could regulate gene expression through sponging miRNA in HCC (Lu *et al*., 2017; Zhu *et al*., 2017). We identified 47 miRNA interacting with both SOX9‐AS1 and SOX9 through bioinformatics tools and picked the five most downregulated miRNA in order to investigate their interaction with SOX9‐AS1. Results suggested that miR‐5590‐3p had the strongest interaction with SOX9‐AS1. Previously, a study showed that miR‐5590‐3p was a tumor‐suppressor gene in gastric cancer (Wu *et al*., 2018). In our study, we initially uncovered the downregulation of miR‐5590‐3p in HCC and confirmed that SOX9‐AS1 regulated SOX9/Wnt/β‐catenin through miR‐5590‐3p. In addition, we found that mutating the miR‐5590‐3p sites on SOX9‐AS1 could partly counteract the inductive effect of SOX9‐AS1 overexpression on SOX9/β‐catenin axis, suggesting that other miRNA might also participate in the regulation of SOX9‐AS1 on the SOX9/Wnt/β‐catenin axis. Moreover, we found using the JASPAR tool that SOX9 potentially targeted SOX9‐AS1 promoter as a TF. Former studies have reported that SOX9 regulated the transcription of lncRNA (Chen *et al*., 2017). Accordingly, we confirmed the transactivation of SOX9‐AS1 by SOX9 and suggested that SOX9‐AS1/miR‐5590‐3p/SOX9 was a positive feedback loop. Rescue assays indicated that SOX9‐AS1 regulated HCC progression through SOX9 and the Wnt/β‐catenin pathway. Finally, *in vivo* assays confirmed that SOX9‐AS1 facilitated tumor growth and metastasis of HCC.

## 5 Conclusion

Our study demonstrated that the SOX9‐AS1/miR‐5590‐3p/SOX9 positive feedback loop drives tumor growth and metastasis in HCC through the Wnt/β‐catenin pathway. SOX9‐AS1 promoted proliferation, migration and invasion of HCC cells *in vitro* and aggravated tumorigenesis of HCC *in vivo*. SOX9‐AS1 sequestered miR‐5590‐3p to upregulate SOX9 and activate the Wnt/β‐catenin pathway. These findings suggest SOX9‐AS1 as a novel prognostic marker and treatment target for HCC.

## Conflict of interest

The authors declare no conflict of interest.

## Author contributions

WZ and YW designed the project; BH and YW acquired the data; DD and ZF analyzed and interpreted the data; ZX wrote the paper.

## Supporting information


**Fig. S1.** Transfection efficiency of SOX9‐AS1 and miR‐5590‐3p. (A) RT‐qPCR results of SOX9‐AS1 level in Huh7 cells with the transfection of sh‐NC or sh‐SOX9‐AS1#1/2/3, and in Hep3B cells with the transfection of pcDNA3.1 or pcDNA3.1/SOX9‐AS1 (named SOX9‐AS1). (B) RT‐qPCR results of miR‐5590‐3p level in Huh7 cells transfected with NC mimic or miR‐5590‐3p mimic (named miR‐5590‐3p), and in Hep3B cells transfected with NC inhibitor or miR‐5590‐3p inhibitor. Significance was determined by Student's t‐test. Error bars indicate SD. All experiments were conducted three times.

**Fig. S2.** Raw data of western blot results related to Figs 1B, 1H, 2H, 4J, 4M, 6B and 7F.
