# Supplementary figures and images for: A SOX9‐AS1/miR‐5590‐3p/SOX9 positive feedback loop drives tumor growth and metastasis in hepatocellular carcinoma through the Wnt/β‐catenin pathway
Source: Mol Oncol. 2019 Aug 31;13(10):2194–210. doi: 10.1002/1878-0261.12560 (PMC6763786; doi:10.1002/1878-0261.12560)

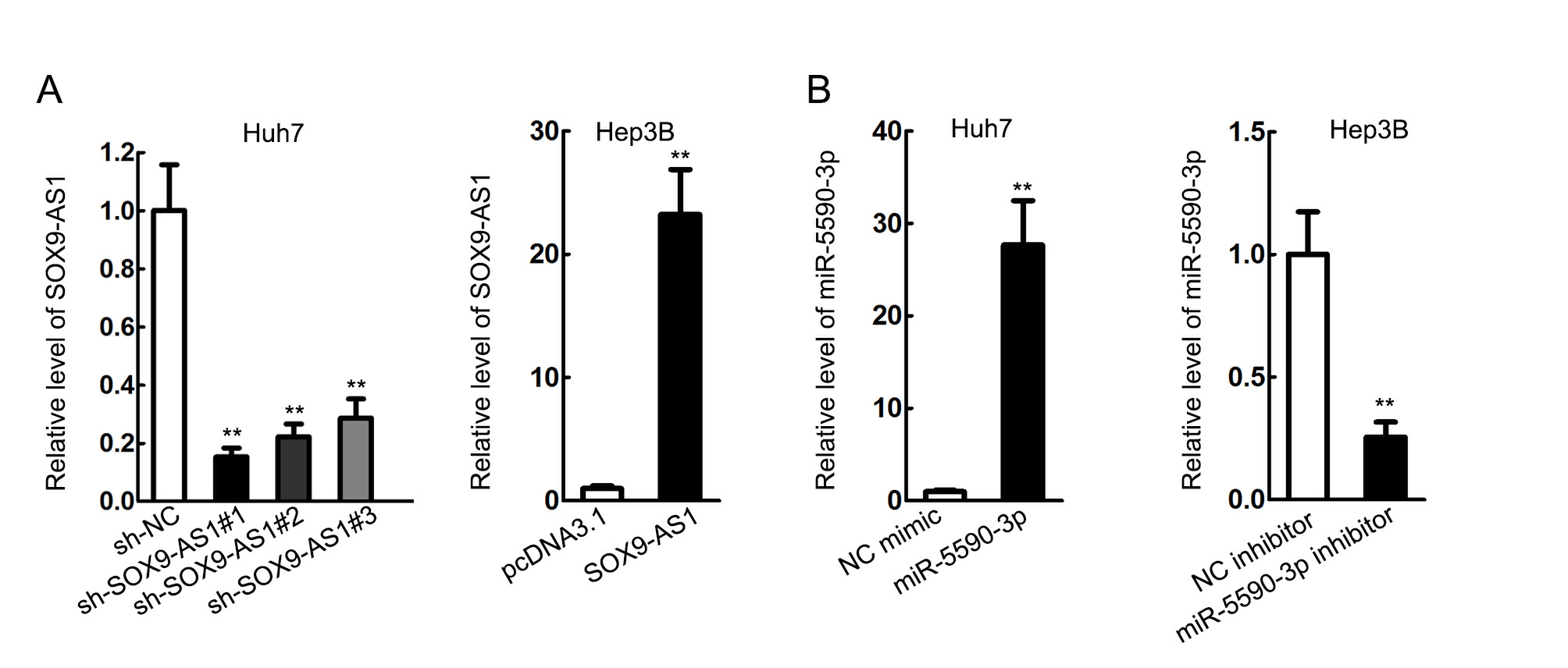

Supplement: Supplementary file 1 — Fig. S1. Transfection efficiency of SOX9‐AS1 and miR‐5590‐3p. (A) RT‐qPCR results of SOX9‐AS1 level in Huh7 cells with the transfection of sh‐NC or sh‐SOX9‐AS1#1/2/3, and in Hep3B cells with the transfection of pcDNA3.1 or pcDNA3.1/SOX9‐AS1 (named SOX9‐AS1). (B) RT‐qPCR results of miR‐5590‐3p level in Huh7 cells transfected with NC mimic or miR‐5590‐3p mimic (named miR‐5590‐3p), and in Hep3B cells transfected with NC inhibitor or miR‐5590‐3p inhibitor. Significance was determined by Student's t‐test. Error bars indicate SD. All experiments were conducted three times. [file MOL2-13-2194-s001.tif]

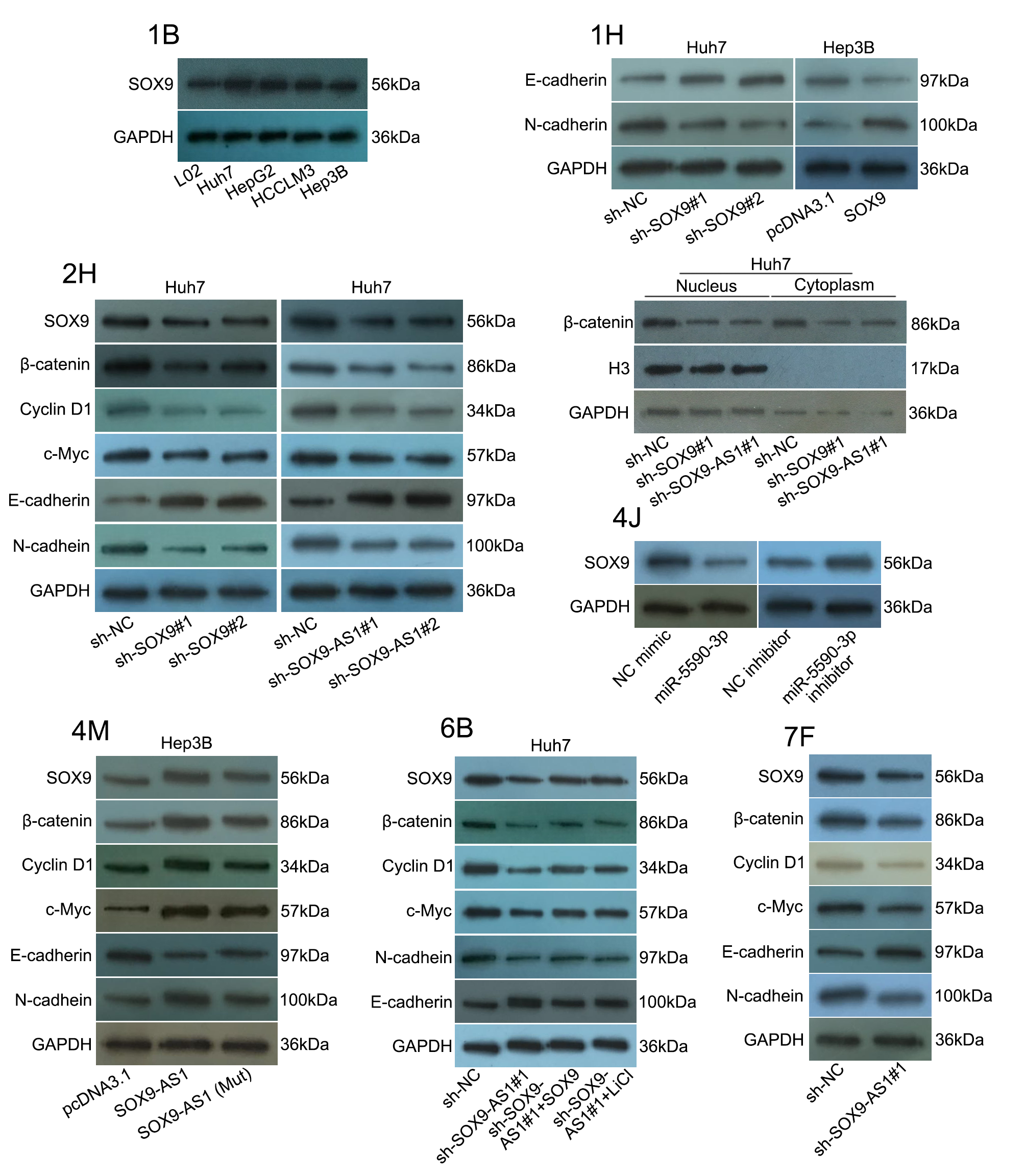

Supplement: Supplementary file 2 — Fig. S2. Raw data of western blot results related to Figs 1B, 1H, 2H, 4J, 4M, 6B and 7F. [file MOL2-13-2194-s002.tif]
